# MAP3K1-targeting therapeutic artificial miRNA suppresses the growth and invasion of breast cancer in vivo *and* in vitro

**DOI:** 10.1186/s40064-015-1597-z

**Published:** 2016-01-04

**Authors:** Chun Liu, Shengjie Wang, Shunxing Zhu, Haifeng Wang, Jiayi Gu, Zeping Gui, Jin Jing, Xiaofan Hou, Yixiang Shao

**Affiliations:** Laboratory animal center of Nantong University, 19 Qixiu Road, Nantong, Jiangsu People’s Republic of China; Kangda College of Nanjing Medical University, 88 Chunhui Road, Lianyungang, Jiangsu People’s Republic of China

**Keywords:** Breast cancer, MAP3K1, Artificial miRNA, Tumor growth, Metastasis

## Abstract

Recent investigations have highlighted that therapeutic artificial microRNAs could be promising candidates for cancer therapy through the modulation of tumor promoter or suppressor. MEK kinase 1 (MEKK1) is expressed by mitogen-activated kinase kinase kinase 1 (MAP3K1), an important kinase that links Ras activation to MAPK signaling. In the present study, we showed that synthetic MAP3K1-targeting artificial miRNA may provide considerable beneficial effects in the prevention of breast cancer growth and metastasis. We showed that MEKK1 was highly expressed in human breast cancer specimens, compared with adjacent normal tissues. Using a miRNA-expressing lentivirus system, we delivered a artificial miRNA (Map3k1 amiRNA) that targets MAP3K1 into 4T1 breast cancer cells and investigated the impact of MAP3K1-targeting miRNA on the growth and invasive behavior of breast cancer in vitro and in vivo. We found that overexpression of Map3k1 amiRNA led to impaired activities of p-ERK and p-p38. In addition, Map3k1 amiRNA induced marked proliferative impairment and invasive attenuation in breast cancer cells. However, Map3k1 amiRNA did not have evident influence on the apoptotic response of 4T1 cells. Moreover, using in vivo nude mice model, we identified that Map3k1 amiRNA attenuated tumor growth and lung metastasis of breast cancer cells. Taken together, our findings explicitly indicated that MEKK1 exerted important oncogenic property in breast cancer development, and MAP3K1-targeting artificial miRNA may provide promising therapeutic effects in the treatment of breast cancer.

## Background

Breast cancer represents the most frequent cancer among women and a leading cause of cancer-associated mortality around the world. It is estimated that there are annually 1.7 million breast cancer cases worldwide, with 522,000 deaths (Ferlay et al. 2015). Despite the fact that vigorous investigations have been conducted by both basic scientists and clinical researchers, the prognosis of breast cancer remains unfavorable, mainly attributed to late diagnosis and limited therapeutic options (Farazi et al. 2013). In addition, the adverse effects of existing therapies restrict their clinical merits in patients (Cuzick et al. 2011). Currently, a promising strategy of breast cancer therapy is to develop molecular target therapy, which has been proved to exert significant beneficial effects in breast cancer treatment. However, the molecular mechanisms underlying breast cancer development remains not completely understood. Therefore, the clarification of therapeutic targets would benefit the development of novel breast cancer therapies.

Breast tumorigenesis involves complex molecular mechanisms. A variety of important cellular signaling pathways have been critically implicated in the initiation and progression of breast cancer, such as MAPK cascades, Wnt/β-catenin, PI3K/AKT etc. From their discoveries, MAPK signaling has been a focus of cancer investigations, including breast cancer (Wagner and Nebreda 2009). MAPKs are a family of typical serine-threonine kinases and are divided into three major MAPK subfamilies, namely p38, JNK and ERK kinases. MAPKs are typical kinase members that are activated through upstream kinase cascades. MEKK1 was initially identified as a kinase that facilitates the activation of MEK and MAPK kinases (Yan et al. 1994). Later on, it was reported that MEKK1 may phosphoryate IκBα and IκBβ kinase complexes, leading to consequent NF-κB activation (Lee et al. 1998). M Russell reported that MEKK1 interacted directly with Ras, implying that MEKK1 may be a downstream effector of Ras signaling (Russell et al. 1995). Accumulating data have suggested a crucial involvement of MEKK1 in tumor biology. For example, blockage of MEKK1 inhibited the survival, invasion and migration of human pancreatic cancer cells (Hirano et al. 2002; Su et al. 2009). Lysophosphatidic acid promotes ovarian cancer cell migration through Ras-MEKK1 signaling (Bian et al. 2004). Rangaswami H reported that Osteopontin triggered the growth and lung metastasis of melanoma via MEKK1-dependent MMP-9 expression (Rangaswami and Kundu 2007). Interestingly, mammary MEKK1-deficient mice exhibit no difference in tumor initiation. However, the dissemination and metastasis of breast cancer is significantly delayed in MEKK1-deficient mice (Cuevas et al. 2006). These findings conceivably indicate that MEKK1 plays a facilitating role in the development of diverse tumor types. However, the expression pattern and physiological significance of MEKK1 in human breast cancer remains to be elucidated.

Recent investigations have pointed to key roles of microRNAs (miRNAs) in the regulation of cellular signaling and cancer biology (Farazi et al. 2013; Miska 2005). It has been well-established that miRNAs modulates the expression of various signaling transducers through binding to their mRNA 3′-terminal and inducing rapid decay of the mRNAs. As such, the utility of synthetic miRNAs as candidate targeted therapy has been proposed as a promising therapy of human diseases (Garofalo and Croce 2011). Kota J reported that the delivery of synthetic therapeutic miR-26a induces cell-cycle arrest in liver cancer cells through targeting Cyclin D2 and E1. Notably, they uncovered that in vivo systemic administration of miR-26a hindered tumor growth, induced tumor-specific apoptosis and protected mice from hepatocellular carcinoma progression with no cytotoxic effects (Kota et al. 2009). In addition to endogenous miRNA, artificially designed miRNAs that specifically target known oncogenes have attracted significant research attention. Masashi Idogawa reported that combined expression of p53 and artificial p21-targeting miRNA induced apparent apoptosis of colon cancer cells in vivo (Idogawa et al. 2009). The delivery of synthetic miRNAs has reportedly exerted powerful tumor-suppressive effects in many other cancer types, such as gastric cancer, bladder cancer and melanoma (Li et al. 2006; Liu et al. 2012a, b). In breast cancer, it was also found that targeting CXCR4 using artificial miRNA blocked the invasion and metastasis of breast cancer cells (Liang et al. 2007). Artificial miRNA-mediated MTDH knockdown inhibits the proliferation, motility and migration of breast cancer cells (Wang et al. 2012). These findings together suggest that artificially designed miRNA may be a valuable approach in the treatment of breast cancer.

Given the information mentioned above, we analyzed the expression of MEKK1 in human breast cancer specimens, and designed an artificial miRNA to specifically suppress the expression of MEKK1 and investigated the impact of miRNA-mediated MAP3K1 silencing on breast cancer physiology. We found that MEKK1 was highly expressed in breast cancer tissues, compared with adjacent normal ones. Moreover, we revealed that blockage of MEKK1 using artificial Map3k1 amiRNA suppressed the growth, lung metastasis and invasion of breast cancer. Our findings implicated that MAP3K1-targeting miRNA might be of important therapeutic merit in breast cancer management.

## Methods

### Cell lines and transfection

Murine breast carcinoma 4T1 cells and Human breast cancer cell lines, MCF-7 and MDA-MB-231 were purchased from ATCC. The cells were maintained in RPMI-1640 medium (Gibco) supplemented 10 % fetal bovine serum (Hyclone). Cells were grown at 37 ℃ in a humidified incubator with 5 % CO_2_ atmosphere. Cells were grown at an exponential growth rate and harvested using 0.1 % trypsin–EDTA when cultures are approximately 80 % confluent. Cells were transfected using Lipofectamine 2000 (invitrogen) according to the manufacturer’s instruments.

### The design of MAP3K1-targeting amiRNA

MAP3K1 gene silencing was achieved by using a vector-based system to produce artificial miRNA. The amiRNA expression vector (pcDNA™6.2-GW/EmGFP-miR) was a gift from Dr. Sun HuaiChang, yangzhou university. We got the sequence of mice MAP3K1 gene from NCBI database (Accession number:NM_011945.2), with the help of INVITROGEN Block-iT RNAi Designer software, we analysed the MAP3K1 sequence and synthesis five candidate miRNA sequences with the highest star-rating (data not shown). Furthermore, Map3k1 amiRNA sequence was subcloned into the eGFP-GV273 vector and lentivirus was packed using the Lentivector Expression Systems (GENECHEM, Shanghai, China).

### Quantitative real time PCR analysis

Total RNA was isolated from breast cancer cells by using Trizol (invitrogen), and cDNA was generated by reverse transcription system kit (invitrogen). Real-time PCR was used to detect relative gene expression using SYBR-Green real-time PCR mixes (Roche). GAPDH was used as the internal control, and the expression difference of MAP3K1 gene between every groups was analyzed by statistical software Graphpad prism 5.

### Western blot analysis

Total protein was isolated from breast cancer cells using RIPA buffer containing 1 % PMSF, protein concentration was determined using a BCA protein assay kit (Beyotime, China). Protein samples were run in a 6 % seperating gel, and then transferred onto a nitrocellulose membrane. The membranes were blocked for 2 h at 4 ℃ with Tris-buffered saline-Tween 20 (TBST) containing 5 % skimmed milk, and probed with primary antibodies angainst MEKK1 (Santa Cruz), ERK and P-ERK (Cell signaling technology, CST), JNK and P-JNK (CST), P38 and p-p38 (CST), and internal control GAPDH (Santa Cruz). Antibodies were diluted in TBST containing 5 % skimmed milk. After 12 h incubation at 4 ℃, the membranes were washed in TBST 10 min for three times. Then, the membrane was incubated with Goat Anti-Rabbit IRDye^®^800CW (ODYSSEY) for 2 h at room temperature. After additional washes, the protein bands were visualized by ODYSSEY System.

### Immunofluorescence cell staining

The cells growing on the glass slide were fixed by 4 % paraformaldehyde, washed in PBS for 20 min, and then permeablized in 0.1 % Triton X-100/TBS for 10 min. Thereafter, the cells were blocked using 10 % goat serum for 1 h. After washing, slides were incubated with antibodies to MEKK1(Santa CRUZ, 1:200) overnight at 4 ℃. After washing, the cells were incubated with Cy3-conjugated anti-goat antibodies (Jackson ImmunoResearch) and 4,6-diamidino-2-phenylindole (DAPI) for 2 h. Images were captured using a Zeiss Axiovert microscope. EdU (Ribobio) and TUNEL (Promega) assays were performed according to manufacturers recommendations.

### H&E and immunohistochemistry

Mammary and lung tissues were fixed in 4 % paraformaldehyde and embedded in paraffin for sectioning. Next, the sections were subjected to HE staining in accordance with the standard HE protocol. Breast cancer clinical pathologic specimens (provided by AFFINIATED HOSPITAL OF NANTONG NUNIVERSITY) were immunostained with an anti-MEKK1 antibody (Santa Cruz, 1:100). Then, SP immunohistochemical staining kits was used according to manufacturers protocol (ZSGB-BIO).

### Migration and invasion assay

Wound-healing experiment was employed to detect the migration ability of 4T1 cells in vitro. 4T1 cells were transfected with Map3k1 amiRNA-3 or contol-amiRNA. When the cells reached 60 % confluence, 1 ml tip was used to scratch about 2 mm region along the plate Central line and the medium was replaced into serum-free medium. The cells were incubated at 37 ℃ for an additional 24 h. The images of cells were capture by Zeiss Axiovert microscope. The number of cells migrated to scratch region were analyzed to compare the migration ability of 4T1cells.

The invasion ability of 4T1 cells was assessed by Transwell (Corning) smeared with Matrigel (BD) assay. 4T1 cells transfected with Map3k1 amiRNA-3 or contol amiRNA were cultivated in serum-free medium, wherein 5 × 10^5^ cells were planted to invade through a filter (8 μm) toward 5 % serum-containing medium for 24 h. Thereafter, the invading cells were stained with Wright’s stian and counted for comparing the invasion ability of 4T1 cells.

### Colony formation assay

A suspension of 4T1 cells (5 × 10^3^ cells) was prepared in 0.35 % agar, which were added to a 6 cm plate over a base agar layer (0.6 %). Overlayed agar changed once a week, plates were incubated at 37 ℃ in a humidified incubator for 2 weeks. At termination, the colonies were stained with Crystal Violet and counted on a dissecting microscope. Positive colonies were defined as clusters consisting of more than 50 cells.

### Tumorigenesis and lung metastasis model

BALB/c-nude mice were employed to analyze the ability of tumorigenesis and lung metastasis of 4T1 cells in vivo. For assessing tumor incidence and growth, 4T1 cells (1.5 × 10^6^ cells/mice) infected with control-amiRNA or Map3k1 amiRNA-3 lentivirus were injected into cleared fat pads of BALB/c-nude mice. Mice were examined for palpable tumors every 2 days, and the growth of the primary tumors was measured once a week using a vernier caliper. Two perpendicular diameters, termed length (L) and width (W), were determined, with length defined as the larger of the two measurements. Volume was calculated using the formula: 1/2 × *l* × W^2^, Mice were euthanized 4 weeks after injection of 4T1 cells. Primary tumors were removed and weighed. H&E staining was performed to determine the pathological structure of primary tumors.

To assess the impact of MAP3K1-targeting miRNA on the metastasis of breast cancer, 4T1 cells (5.0 × 10^5^ cells/mice) infected with control-amiRNA or Map3k1 amiRNA-3 lentivirus were transplanted into mice by tail vein injection. The weight of mice was measured once a week. Mice were euthanized 4 weeks after injection of 4T1 cells. Metastasis was evaluated in lungs, which is typical for this tumor due to their visibility and ease of quantification. Lungs were fixed in Bouin’s solution, and metastases were quantified by counting on a dissecting microscope. Finally, H&E staining was performed to analyze the pathological structure of lung metastasis of breast cancer.

### Statistics

Data were analyzed using Prism software (GraphPad Software, Inc., La Jolla, CA). Values were compared using a student’s *t* test, and a two-sided *p* value of ≤0.05 was considered significant, and ≤0.01 means very significant.

## Results

### MEKK1 was highly expressed in human breast cancer specimens

**Fig. 1:**
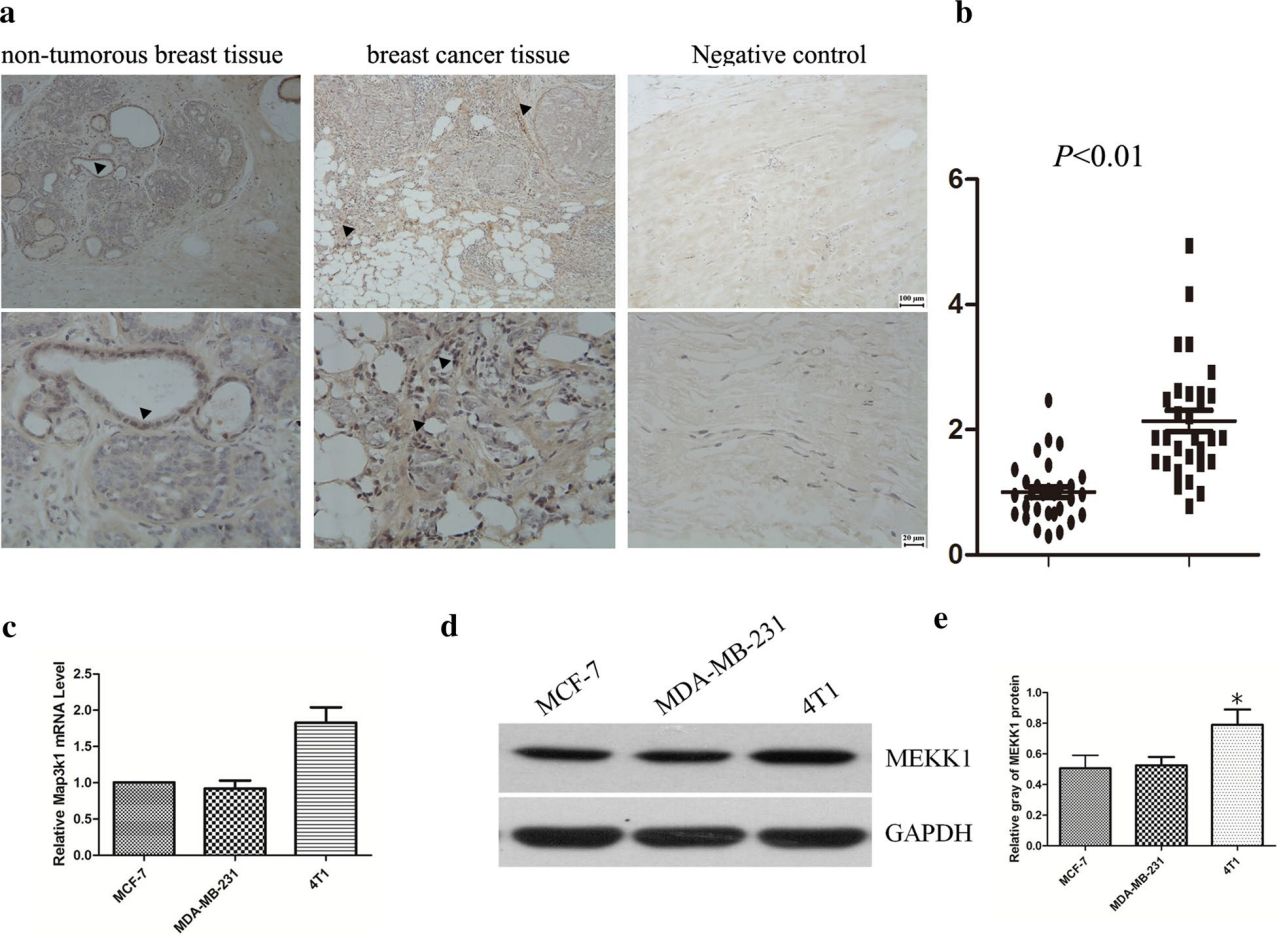
MEKK1 was highly expressed in invasive breast cancer specimens and breast cancer cell lines. **a** Representative images of MEKK1 expression in breast cancer specimens and adjacent normal tissues. upper panel, 20× magnification; lower panel, 40× magnification. **b** Statistical analysis of MEKK1 expression in breast cancer specimens and adjacent normal tissues. **c** Real-time PCR analysis of MAP3K1 mRNA expression in breast cancer cell lines. **d** Western blot analysis of MEKK1 expression in different breast cancer cell lines. **e** The bar chart indicates the ratio of MEKK1 protein level to glyceraldehyde 3-phosphate dehydrogenase (GAPDH) by densitometry in breast cancer cells (*p < 0.05)

To explore the involvement of MEKK1 in breast cancer development, the expression pattern of MEKK1 was first determined using immunohistochemical analysis. As shown in Fig. 1a, b, the expression of MEKK1 was markedly upregulated in breast cancer tissues, compared with adjacent non-tumorous ones. Notably, MEKK1 was especially abundantly expressed in invasive breast cancer samples. Next, we analyzed the expression of MEKK1 in different breast cancer cell lines. Real-time and western blot analysis indicated that MEKK1 was also enriched in breast cancer cell lines, particularly in marine 4T1 breast cancer cells (Fig. 1c–e). Therefore, these data implied that MEKK1 might exert a facilitating role in breast carcinogenesis.

### Overexpression of Map3k1 amiRNA down-regulated ERK signaling in breast cancer cells

**Fig. 2:**
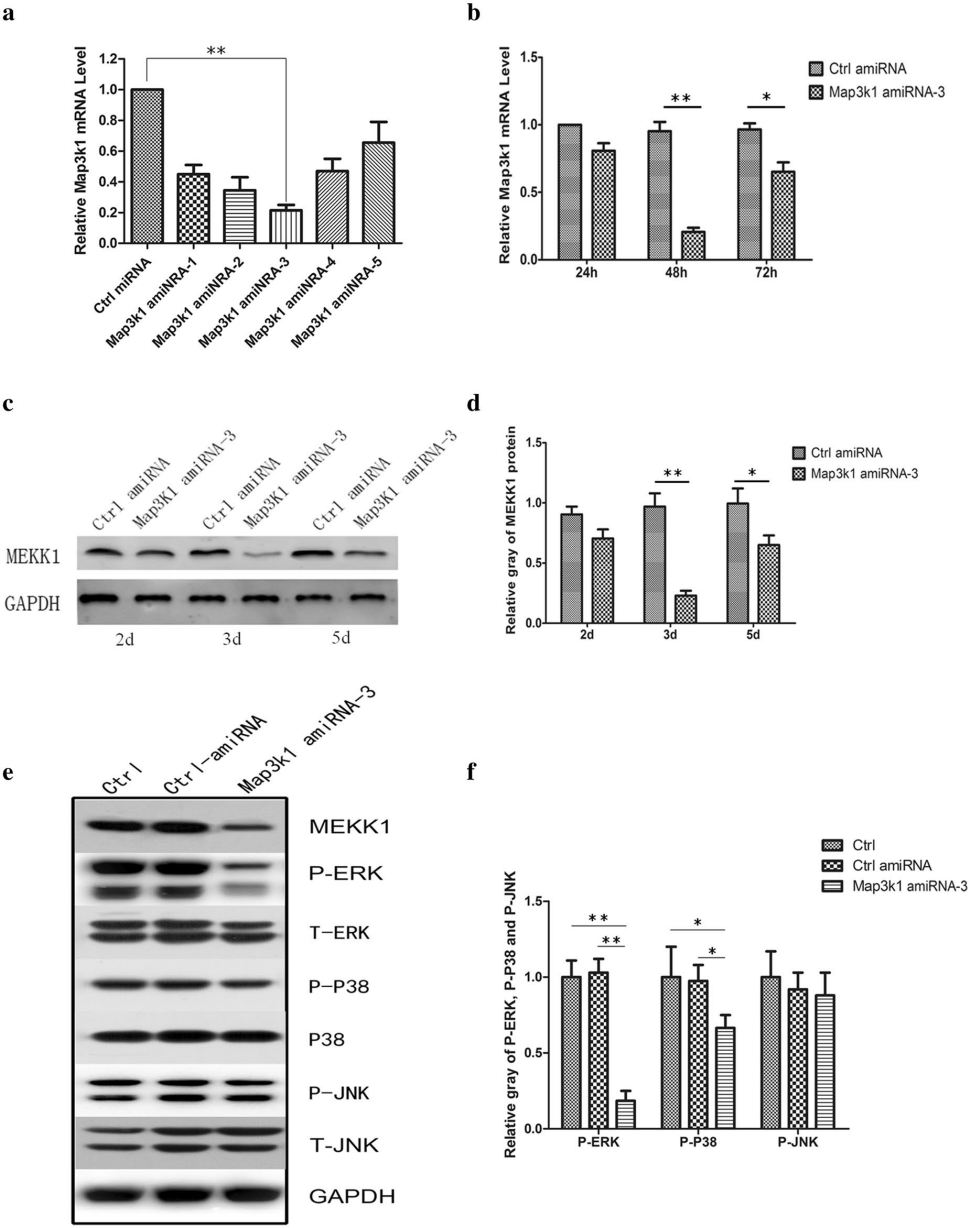
Transfection of Map3k1 amiRNA significantly impaired cellular level of MEKK1 and downstream MAPK pathways in breast cancer cells. **a** Real-time PCR analysis of the interference efficiencies of different Map3k1 amiRNA constructs. **b** Time-dependence of MAP3K1 mRNA expression following Map3k1 amiRNA transfection using real-time PCR analysis. **c** Western blot detection of MEKK1 expression after Map3k1 amiRNA transfection. **d** The *bar chart* indicates the ratio of MEKK1 protein expression to GAPDH using densitometry after Map3k1 amiRNA transfection. **e** Determining the influence of Map3k1 amiRNA-3 overexpression on the levels of p-ERK, p-p38 and p-JNK MAPKs using western blot analysis. **f** Quantitative analysis of the intensities of MAPK protein bands relative to GAPDH in the indicated groups (*p < 0.05, **p < 0.01)

Next, we investigated the role of MEKK1 in the regulation of breast cancer growth and invasion. Because recent studies highlighted that artificial miRNA could effectively target genes to modulate the physiology of cancer cells, we designed 5 MAP3K1-targeting miRNAs using Invitrogen Block-iT RNAi Designer program, and constructed the precursors of the 5 miRNAs into pcDNA™6.2-GW/EmGFP-miR vector. The constructs have been verified using PCR analysis and DNA sequencing (Data not shown). Thereafter, the constructs were transfected into 4T1 cells. Using real-time PCR analysis, we found that Map3k1 amiRNA-3 achieved the best interference efficiency (Fig. 2a). As predicted, the protein level of MEKK1 was also dramatically down-regulated after the transfection of Map3k1 amiRNA-3 (Fig. 2b–d). Because MEKK1 was documented to activate MAPKs, we detected the level of phosphorylated p-38, JNK and ERK in 4T1 cells transfected with control-amiRNA or Map3k1 amiRNA-3. As shown in Fig. 2e, f, the transfection of Map3k1 amiRNA-3 decreased the level of phosphorylated ERK (p-ERK), and to a less extent, p-38. However, we did not observe any alterations in the level of phosphorylated JNK in the cells. These findings suggested that artificial miRNA-mediated deprivation of MEKK1 could selectively inhibit ERK and p-38 signaling in breast cancer cells.

### Map3k1 amiRNA attenuated the proliferation of 4T1 breast cancer cells

**Fig. 3:**
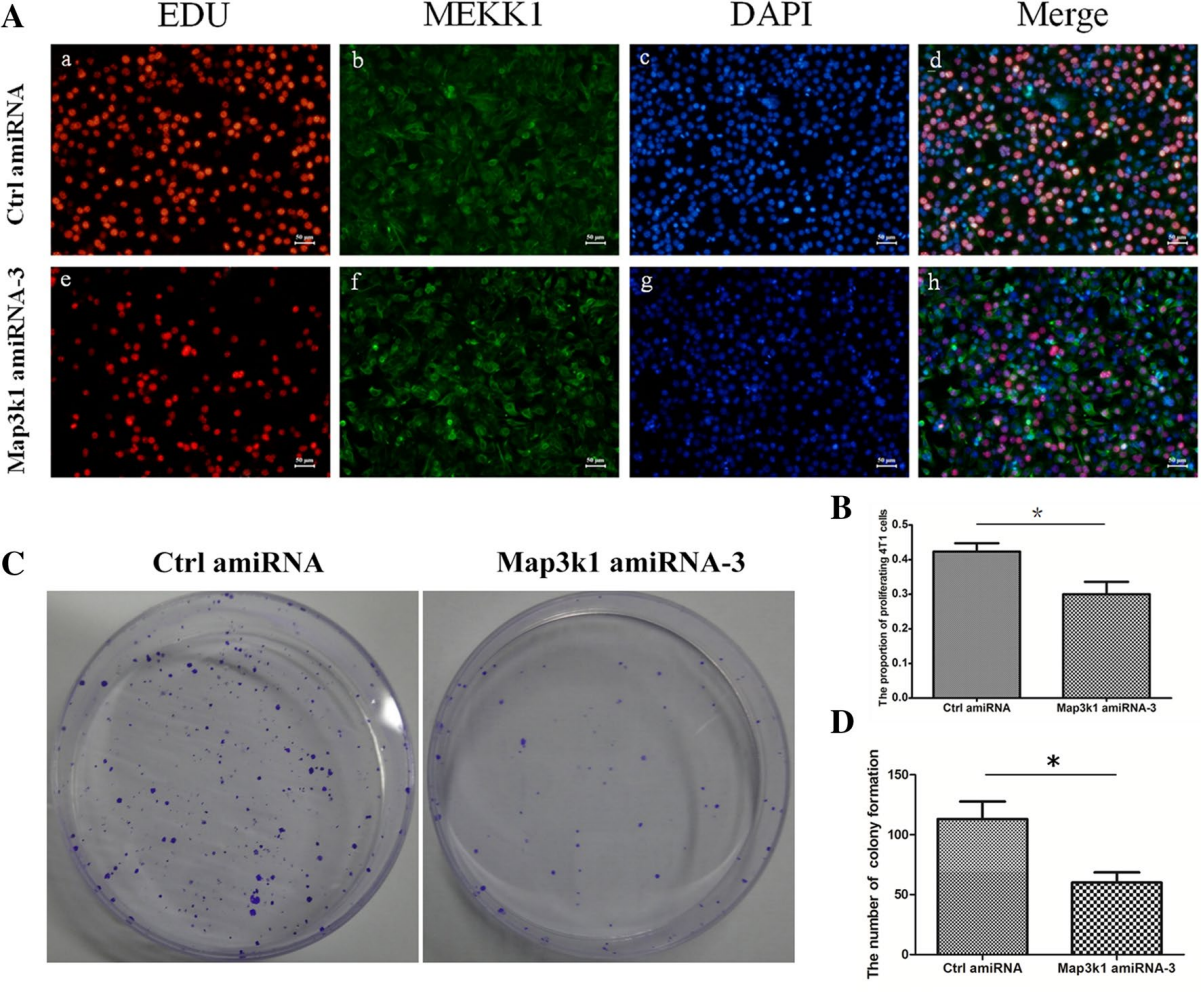
The influence of Map3k1 amiRNA overexpression on the proliferation of 4T1 breast cancer cells. **A** After Lentivirus infection, control-amiRNA and Map3k1 amiRNA-overexpressing 4T1 cells were subjected to EDU incorporation assay. **B** Statistical analysis of EDU-positive cells in control-amiRNA and Map3k1 amiRNA-overexpressing cells (*p < 0.05). **C** Representative images of the colony formation of control-amiRNA and Map3k1 amiRNA-overexpressing cells in vitro. **D** Statistical analysis of colony formation capacities of control-amiRNA and Map3k1 amiRNA-overexpressing 4T1 cells (*p < 0.05)

Because ERK and p-38 signaling have been documented to regulate the growth and invasion of breast cancer cells, we analyzed whether transfection of Map3k1 amiRNA-3 could influence the growth of breast cancer cells. To this end, we constructed Map3k1 amiRNA-3 precursor into a Lentivirus construct. Thereafter, Map3k1 amiRNA Lentivirus was obtained by co-transfection of the Map3k1 amiRNA-3 lentivirus and helper vectors. 4T1 cells were infected with control virus or Map3k1 amiRNA virus and subjected to real-time PCR analysis to determine the level of MAP3K1 mRNA in the cells, which indicated that the level of MAP3K1 mRNA was apparently decreased after the infection of Map3k1 amiRNA virus (Data not shown). Next, the cells were subjected to EDU incorporation assay to determine the proliferation of the cells. As shown in Fig. 3A, B, the rate of EDU incorporation declined remarkably after Map3k1 amiRNA transduction, suggesting that Map3k1 amiRNA significantly impaired the proliferation of 4T1 breast cancer cells. Furthermore, the colony formation assay was performed. Coinciding with the reduced EDU rate, the colony formation capacities of Map3k1 amiRNA-infected cells was evidently impaired, compared with control cells (Fig. 3C, D).

### Overexpression of Map3k1 amiRNA inhibited the migration and invasion of breast cancer cells

**Fig. 4:**
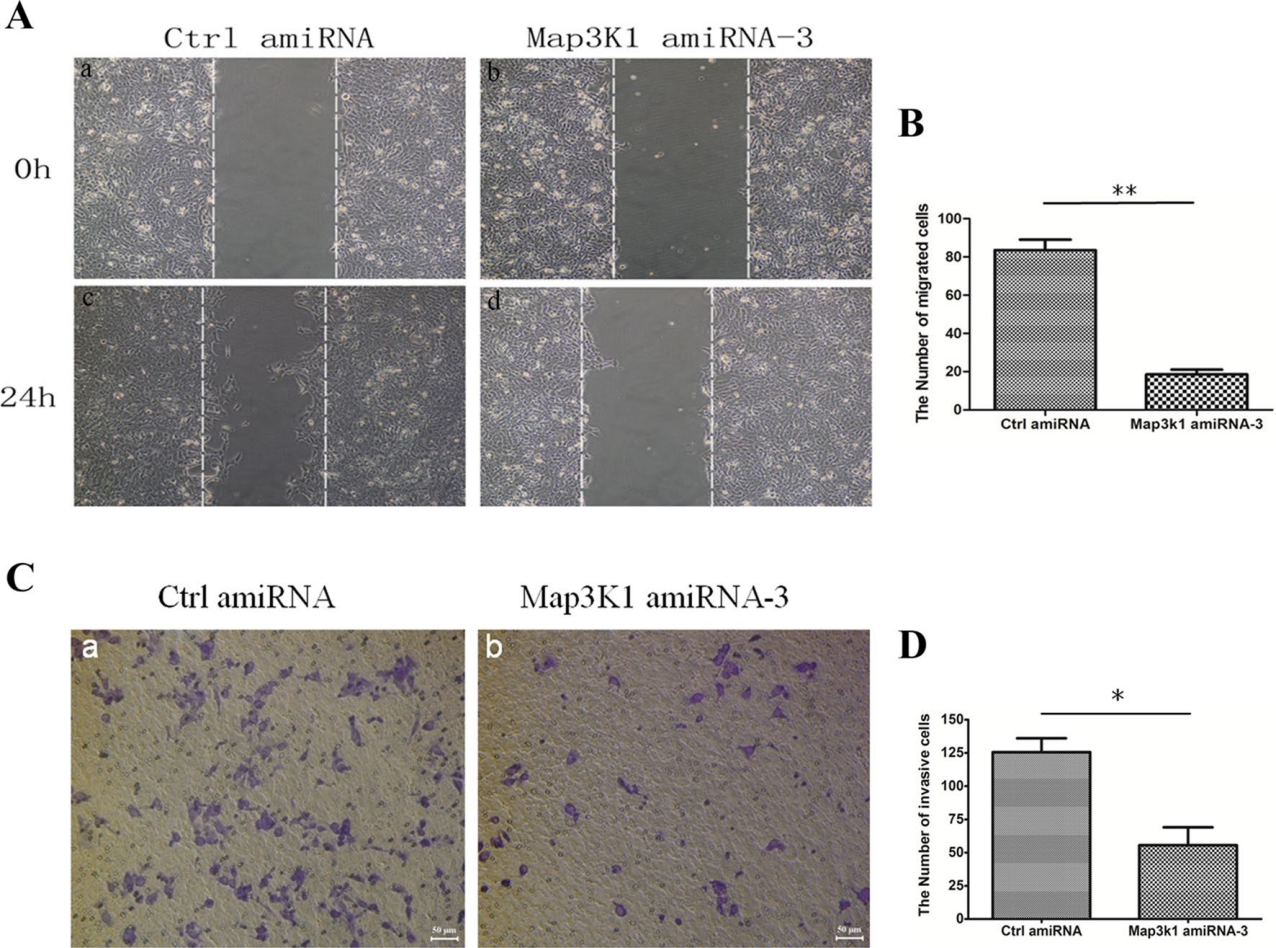
Overexpression of Map3k1 amiRNA impaired the migration and invasion of breast cancer cells. **A** Scratch assay was performed to determine the impact of Map3k1 amiRNA-overexpression on the migration of breast cancer cells. **B**. Statistical analysis of migrated cells during 24 h in both control-amiRNA and Map3k1 amiRNA-overexpressing cells (**p < 0.01). **C** Determination of invasive capacity of control-amiRNA and Map3k1 amiRNA-overexpressing 4T1 cells using Matrigel transwell assay. **D** The *bar chart* indicates the number of invasive cells between control-amiRNA and Map3k1 amiRNA-overexpressing groups (*p < 0.05)

Because ERK and p-38 were reported to play regulatory roles in the invasion of breast cancer cells, we addressed whether overexpression of Map3k1 amiRNA-3 might inhibit the migration and invasion of breast cancer cells (Gomes et al. 2012). Thus, control and Map3k1 amiRNA-3 overexpressing 4T1 cells were subjected to scratch and Matrigel transwell assays. 24 h after scratch, control cells exhibited apparent migration into scratched region (Fig. 4A). However, overexpression of Map3k1 amiRNA-3 significantly impaired the migrating ability of the cells (Fig. 4A, B). As predicted, transwell assay also revealed that Map3k1 amiRNA-3 overexpression attenuated the number of cells that invaded through Matrigel (Fig. 4C, D). Therefore, these results clearly indicated that overexpression of Map3k1 amiRNA-3 impaired the migration and invasion of 4T1 breast cancer cells.

### Overexpression of Map3k1 amiRNA did not affect the apoptotic response of breast cancer cells

**Fig. 5:**
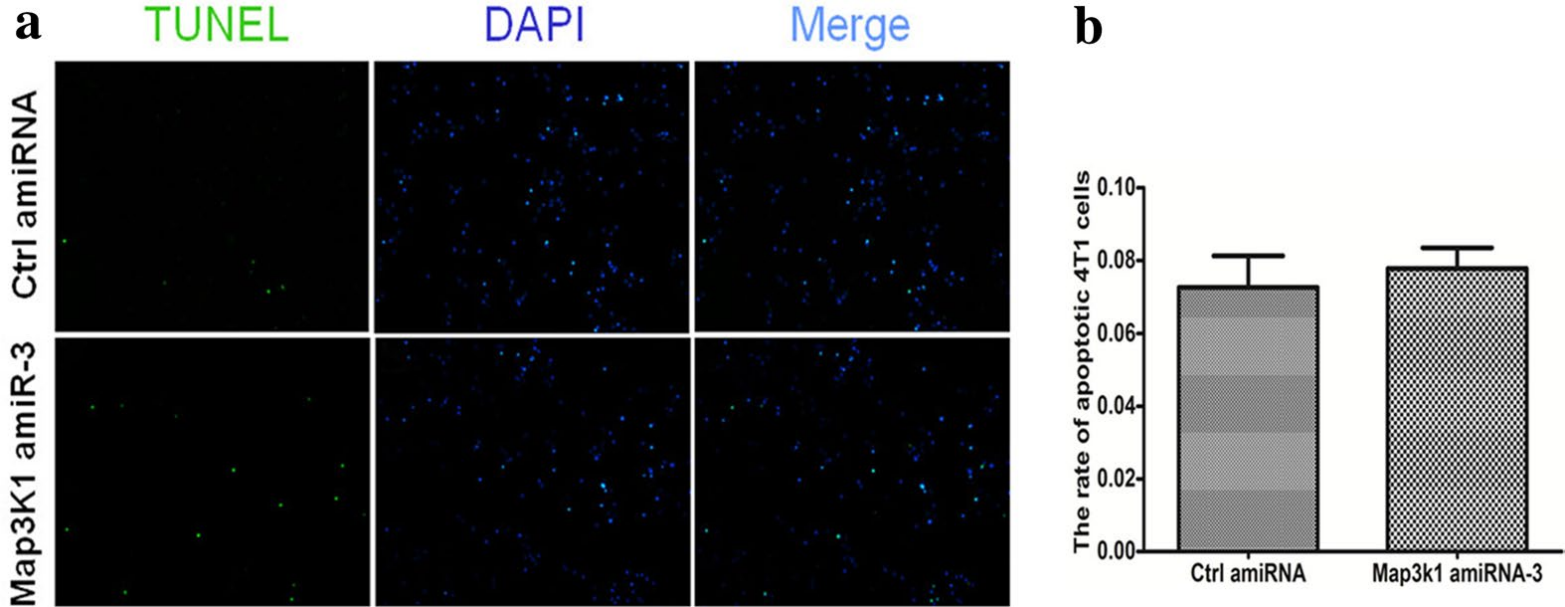
Map3k1 amiRNA did not affect the apoptosis of 4T1 breast cancer cells. **a** TUNEL assay revealed that 4T1 cells exhibited comparable apoptotic rate between control-amiRNA and Map3k1 amiRNA-overexpressing groups. **b** The *bar chart* shows that the apoptosis of cells did not differ statistically between control-amiRNA and Map3k1 amiRNA-overexpressing cells

Furthermore, we analyzed whether the apoptotic response were affected after Map3k1 amiRNA-3 overexpression. TUNEL assay was employed to determine the apoptotic rate of control and Map3k1 amiRNA-overexpressing 4T1 cells. As shown in Fig. 5a, b, the two groups of cells did not exhibit any difference in the apoptotic rate. Thus, Map3k1 amiRNA-3 may not influence the apoptotic response of breast cancer cells.

### Overexpression of Map3k1 amiRNA alters the growth and metastasis of breast cancer cells in vivo

**Fig. 6:**
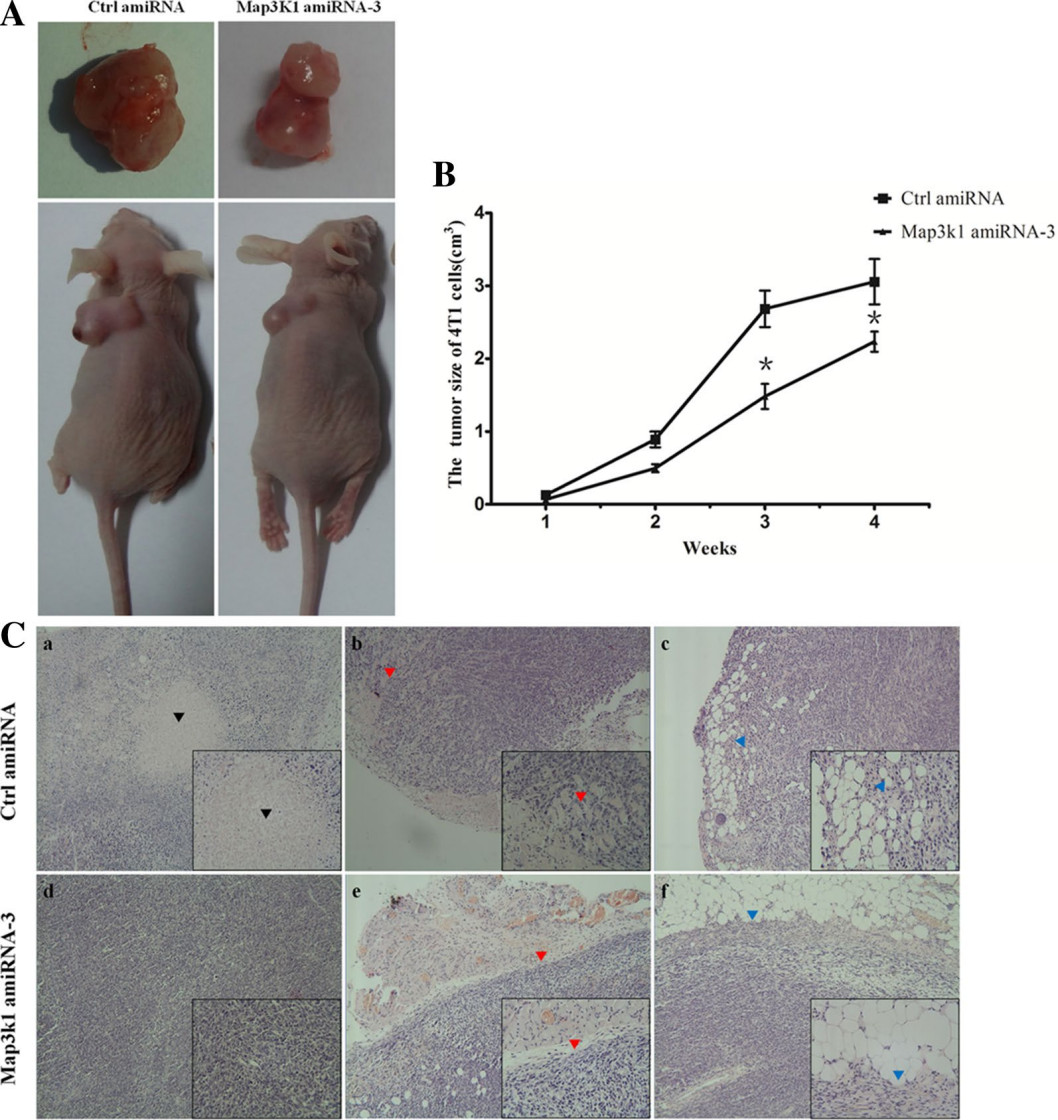
Overexpression of Map3k1 amiRNA attenuated the in vivo tumor formation of 4T1 breast cancer cells. **A** Representative images of tumor formation in control-amiRNA and Map3k1 amiRNA-overexpressing cells in nude mice. **B** The growth curve of control-amiRNA and Map3k1 amiRNA-overexpressing tumors (*p < 0.05). **C** HE staining of control-amiRNA and Map3k1 amiRNA-overexpressing tumors indicated that control tumors displayed necrotic-like structure, invasion into muscle and fat tissues, whereas similar phenomena were not identified in Map3k1 amiRNA-overexpressing tumors (a, *arrowheads* indicated necrotic-like region; b, *arrowhead* indicated tumor invasion into muscle tissues; c, *arrowhead* tumor invasion into fat tissues)

To further delineate the involvement of artificial miRNA-induced MAP3K1 depletion in breast cancer progression, we investigated the in vivo growth and metastasis of breast cancer cells using nude mice model. Control and Map3k1 amiRNA-overexpressing 4T1 cells were injected into the flanks of BALB/c nude mice. The sizes of tumors were measured weekly, and the growth curves of the tumors were produced. As shown in Fig. 6a, b, 4 weeks after injection, the tumor size and weight of control cells were significantly larger than Map3k1 amiRNA-overexpressing cells (Table 1). In addition, the tumors were subjected to HE staining. Map3k1 amiRNA-overexpressing tumors displayed very clear tumor border and regular inner structure. However, in control tumors, necrotic-like regions were frequently observed, probably because the tumors were larger in size and could be easily affected by many micro-environmental factors, such as hypoxia and nutritional deficiency. Of note, control tumors exhibited invasive behaviors into muscle and fat tissues, which were rarely found in Map3k1 amiRNA-overexpressing tumors (Fig. 6C).

**Table 1:** The effect of MEKK1 suppression on tumorigenic capacity of 4T1 cells

Group	Incidence of tumorigenesis	Tumor weight (g)	Tumor size (cm^3^)
Ctrl amiRNA	8/8	5.41 ± 1.39	3.26 ± 0.85
Map3k1 amiRNA-3	7/8	3.09 ± 0.54*	2.35 ± 0.25*

Data showed that tumor size and weight of control cells were significantly larger than Map3k1 amiRNA-overexpressing cells (*p < 0.05)

**Fig. 7:**
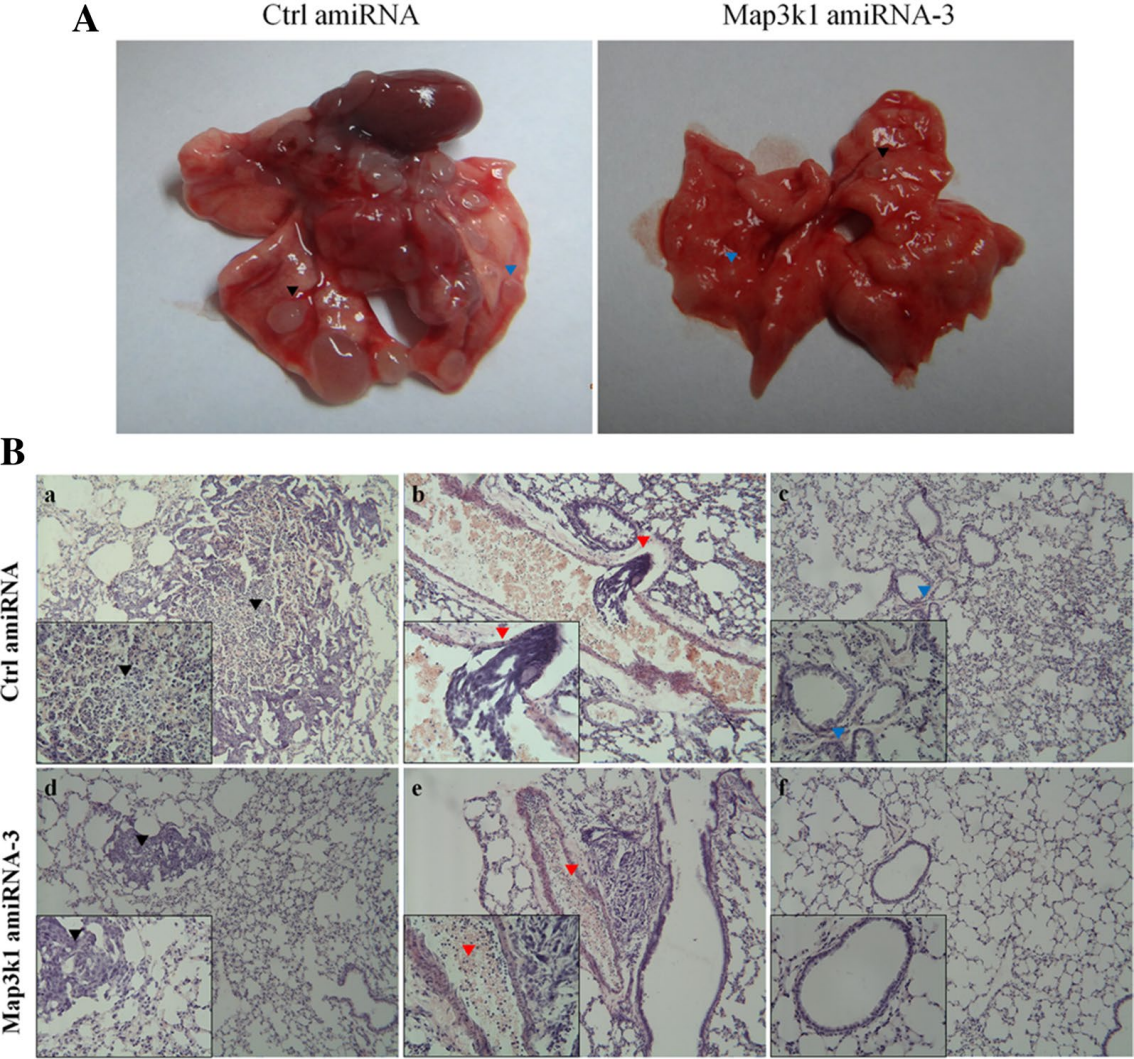
Map3k1 amiRNA reduced lung metastasis of 4T1 breast cancer cells. **A** The formation of lung tumor niches in control-amiRNA and Map3k1 amiRNA-overexpressing 4T1 cells after tail vein injection. Control-amiRNA 4T1 cells formed significantly higher numbers of tumor niches than Map3k1 amiRNA-overexpressing ones (*arrowhead* pointed to tumor niches). **B** HE staining was conducted to verify the tumor niches of the two groups (*a*, *d*, *arrowhead* indicated the region area of tumor niches formed by control cells were obviously larger than Map3k1 amiRNA-overexpressing cells; *b*, *arrowhead* pointed to the metastatic tumor formed by control 4T1 cells in blood vessel of lungs; *c*, *arrowhead* indicated inflammatory reaction and hyperplasia in lung tissue were occurring in control amiRNA group, whereas Map3k1 amiRNA-3 group maintained a integrated lung tissue)

To further elucidate the role of Map3k1 amiRNA in the regulation of breast cancer metastasis, we assessed whether Map3k1 amiRNA-3 could impair the ability of 4T1 cells to colonize the lungs of Balb/c nude mice. 4 weeks after cell injection, the mice were sacrificed and lung metastasis of breast cancer cells was examined. As shown in Fig. 7A, control cells formed many metastatic niches in the lungs of nude mice, whereas mice injected with Map3k1 amiRNA-overexpressing cells had very few tumors in their lungs (Table 2). HE staining analysis also revealed that overexpression of Map3k1 amiRNA-3 reduced the rate of lung metastasis and invasive behavior of breast cancer cells in nude mice xenograft model (Fig. 7B). Taken together, these data indicated that Map3k1 amiRNA-induced MAP3K1 depletion inhibited the growth and lung metastasis of breast cancer cells.

**Table 2:** The effect of MEKK1 suppression on metastatic capacity of 4T1 cells

Group	Death rate	Incidence of metastasis	Nodules/lung
Ctrl amiRNA	4/10	10/10	17 ± 9
Map3k1 amiRNA-3	2/10	8/10	8 ± 5*

Data showed that control-amiRNA 4T1 cells formed significantly higher numbers of tumor niches than Map3k1 amiRNA-overexpressing ones (*p < 0.05)

## Discussion

The MAPK cascades are central signaling pathways that play fundamental roles in many aspects of cellular processes, including proliferation, differentiation, migration and apoptosis. In the present study, we investigated the role and potential therapeutic value of MEKK1 in breast cancer. We found that MEKK1 was upregulated in a majority of breast cancer samples. Of great importance, we showed that miRNA-based MAP3K1 targeting significantly suppressed the proliferation and invasion of breast cancer cells. Additionally, using nude mice, we identified that Map3k1 amiRNA treatment attenuated the progression and lung metastasis of breast cancer in vivo. These data undoubtedly supported the notion that MEKK1 plays an important role in the regulation of breast cancer growth and metastasis, highlighting that miRNA-targeted MAP3K1 depletion is a valuable approach in the treatment of breast cancer.

In recent years, the development of targeted therapy is a most attractive strategy of cancer therapy. In this regard, RNA-based modulation of mRNA and protein expression has attracted significant research attention (Tong et al. 2005). RNA interference has been widely regarded as a promising approach to achieve the goal of protein expression modulation (Devi 2006). However, due to their transient expression and limited delivery system in vivo, the therapeutic efficiencies of RNAi oligos remain unsatisfactory (Zhao et al. 2009). In addition, the use of RNA polymerase III as transcription machinery is difficult to control their expression in desired targeting tissues and cells. miRNAs, a class of polymerase II-directed endogenous small single-stranded RNAs (ssRNA) that share similar acting mechanism with RNAi, may avoid the limitations. It was recently reported that modified shRNA construct using miRNA-based processing machinery may offer more reliable and profound outcome of gene therapy with few side effects in vivo (Stegmeier et al. 2005; McBride et al. 2008). In the current study, we examined whether a similar system may provide favorable result in breast cancer treatment. We found that Map3k1 amiRNA conferred marked tumor-suppressive and anti-metastatic effects through stable MEKK1 depletion in vitro and in vivo. These findings explicitly infer that artificial miRNA may provide considerable therapeutic merit in cancer management.

The oncogenic property of MEKK1 was largely attributed to the activations of downstream MAPK kinases. MAPK kinases play complicated roles in the development of human cancers. For example, ERK MAPKs are activated by a wide range of mitogens and play a predominant role in promoting the growth and metastasis of cancer, including breast cancer (Samatar and Poulikakos 2014). Studies have established that ERK was responsible for enhanced proliferation and migration of breast cancer under many conditions. Bianchi-Smiraglia A et al. reported that integrin β5 contributed to malignant proliferation, tumor angiogenesis and metastasis of breast cancer through the activation of Src-FAK and MEK-ERK signaling pathways (Bianchi-Smiraglia et al. 2013). Discoidin domain receptor 2 (DDR2) facilitates the metastasis of breast cancer through ERK-mediated Snail1 phosphorylation (Zhang et al. 2013). Studies have shown that ERK conferred the proliferation of breast cancer cells through phosphorylation-dependent downregulation and nuclear export of p27 (Foster et al. 2003). In addition to Snail and p27, approximate 160 substrates of ERK kinase have been identified, many of which are critically involved in breast cancer growth and metastasis, such as c-myc, Elk-1 and MSK (Yoon and Seger 2006). These findings implicated an important role of ERK signaling in breast cancer progression and invasion. Coinciding with the notion, our results identified that amiRNA-mediated MAP3K1 silencing could significantly influence ERK pathway, partially accounting for the impaired tumor growth and metastasis. In addition to ERK, our findings suggested that p38 MAPK, to a less extent, was also affected by Map3k1 amiRNA. It is noteworthy that p38 also contributes to the proliferation and metastasis of breast cancer, and inhibition of p38 leads to impaired proliferation of ER-negative breast cancer cells (Chen et al. 2009; Kim et al. 2003). However, some other studies implicated that p38 activation resulted in growth impairment of breast cancer (Cocolakis et al. 2001; Yu et al. 2007). Thus, it remains unclear about the detailed role of p38 in breast cancer physiology following Map3k1 amiRNA overexpression. By contrast, JNK signaling is frequently related with apoptotic response in cancer cells under stress conditions. Our study found that JNK activity was largely unchanged after MEKK1 inactivation, which was consistent with comparable apoptosis between control and Map3k1 amiRNA-overexpressing cells. Notably, as mentioned in introduction section, MEKK1 also played an important role in the regulation of NF-κB signaling, and it is worthy to examine whether NF-κB signaling was altered in our experimental settings, because of the central role of NF-κB signaling in facilitating the metastasis and growth of breast cancer (Huber et al. 2004; Biswas et al. 2004). Taken these information into account, we speculated that Map3k1 amiRNA may decrease the growth and metastasis of breast cancer cells through the regulation of multiple signaling pathways.

## Conclusions

Taken all the experimental data in vitro and in vivo, we for the first time reported that MEKK1 was significantly upregulated in breast cancer specimens. Moreover, our findings supported that synthetic artificial miRNA that targets MAP3K1 exert remarkable growth-inhibitory and anti-metastatic effects in breast cancer cells, highlighting that artificial miRNA may confer significant anti-tumor activity through modulating the expression of oncoproteins.
